# Meningoencephalitis in Neonatal Calves Caused by Rare O161 Serotype *Escherichia coli*: A Case Report

**DOI:** 10.3390/vetsci13101037

**Published:** 2026-10-01

**Authors:** Chen Ling, Aohong Wang, Qiwen Zhang, Chengwu Hao, Sun He, Yu Zhu, Ye Li, Yuxuan Lu, Zhengliang Zhang, Jinglin Li, Genqiang Yan, Jingjing Ren, Jianjun Jiang

**Affiliations:** 1 Tecon Bio-Technology Co., Ltd., Urumqi 830000, China; 2 Engineering and Technology Research Center for Veterinary Biological Products, Xinjiang Production and Construction Corps, Urumqi 830000, China; 3 College of Animal Science and Technology, Shihezi University, Shihezi 832003, China

**Keywords:** calf, meningoencephalitis, *Escherichia coli*, O161 serotype, multidrug resistance, *ibeB*, case report

## Abstract

Meningoencephalitis is a rare but fatal form of colibacillosis in neonatal calves. An outbreak of neurological disease characterized by high fever, lethargy, opisthotonus, and death occurred in approximately one-week-old calves on a dairy farm in Xinjiang, China; 13 calves died despite antibiotic therapy. Necropsy revealed severe meningeal hemorrhage and increased cerebrospinal fluid. Five *Escherichia coli* isolates were obtained from two dead calves (brain-derived CHN-XJ-B1–B4; liver-derived CHN-XJ-L). The isolates carried the K99 and STa virulence genes, and two representative strains were serotyped as the rare O161. All isolates were resistant to 12 of 16 tested antibiotics but susceptible to amikacin, cefazolin, and neomycin. The median lethal doses (LD_50_) in mice were 9.8 × 10^5^ CFU (CHN-XJ-B1) and 2.3 × 10^6^ CFU (CHN-XJ-L). Histopathology showed cerebral vascular congestion, neuronal degeneration and necrosis, and neutrophilic meningeal infiltration. All isolates harbored the invasion gene *ibeB*; this gene was nearly identical to that of the non-pathogenic *E. coli* K-12 strain MG1655 (98.5–100% nucleotide identity) and shared approximately 96% nucleotide identity with the human meningitis strain RS218. Deletion of *ibeB* significantly reduced bacterial burdens in the liver and spleen and in the brain at 48 h post-infection, increased the LD_50_ by approximately tenfold and significantly prolonged mouse survival, indicating that *ibeB* contributes to systemic dissemination and tissue colonization of the isolates in this model. This is the first report of calf meningoencephalitis caused by O161 *E. coli* in the international literature.

## Simple Summary

Neurological disease caused by *Escherichia coli* is rarely seen in newborn calves. Here, we report an outbreak on a dairy farm in Xinjiang, China, in which 13 calves died despite antibiotic treatment. The bacteria isolated from the brain and liver of the dead calves belonged to serotype O161, which has not previously been linked to calf meningoencephalitis in the international literature, and were resistant to most of the antibiotics commonly used on dairy farms. The isolates also carried *ibeB*, a gene previously linked to *E. coli* invasion of brain cells in human meningitis; deleting this gene reduced bacterial burdens in the liver and spleen and in the brain at the later stage of infection, and made the bacteria less deadly in mice. This case highlights the importance of choosing antibiotics on the basis of susceptibility testing and adds O161 to the list of *E. coli* serotypes that can cause meningitis in cattle.

## 1. Introduction

*Escherichia coli* is one of the most important Gram-negative pathogens causing extraintestinal infections in humans and animals. Extraintestinal pathogenic *E. coli* (ExPEC) can asymptomatically colonize the intestinal tract but is able to cause a variety of diseases, including neonatal meningitis and urinary tract infections in humans, septicemia and meningitis in pigs, airsacculitis and septicemia in poultry, and diarrhea, septicemia, and meningitis in calves [1,2,3,4]. Among the ExPEC pathotypes, neonatal meningitis-causing *E. coli* (NMEC) is typified by strains expressing the K1 capsular polysaccharide; these strains disseminate through the bloodstream to the central nervous system and cross the blood–brain barrier (BBB) by interacting with brain microvascular endothelial cells (BMECs) [5,6,7,8]. Bacterial determinants involved in BMEC invasion include the K1 capsule, OmpA, the Ibe protein family (IbeA, IbeB, and IbeC/YijP), and AslA [9,10,11,12,13,14], and the molecular mechanisms by which NMEC causes meningitis remain an active area of research [8].

In cattle, colibacillosis most commonly manifests as neonatal diarrhea or septicemia, whereas the meningitic form is rarely reported. However, recent studies from South America and Asia have confirmed that multidrug-resistant *E. coli* can cause meningitis and septicemia in dairy calves [15,16], indicating that this disease remains a persistent threat to the cattle industry. An outbreak of fatal neurological disease in neonatal calves occurred on an intensive dairy farm in Shihezi, Xinjiang, China. We isolated *E. coli* from the brain and liver tissues of dead calves and characterized its invasion gene *ibeB*, which is highly homologous to that of the human meningitis *E. coli* K1 strain RS218; the cloning and sequence analysis of this gene have been reported in our previous study [17]. Part of the preliminary characterization of these isolates—namely their isolation and identification, including O161 serotyping, and the detection of the virulence genes K99 and STa—has been reported in our previous Chinese-language studies [17,18], and the construction of the *ibeB* deletion mutant was described in a master’s thesis [19]. The present manuscript is the first journal publication of the clinical and pathological documentation of the outbreak, the antimicrobial resistance profiling, and the in vivo functional evaluation of *ibeB*. Here, we report the clinical course, laboratory diagnosis, antimicrobial resistance profile, and pathogenicity of this outbreak, and we evaluated the role of *ibeB* in the pathogenicity of the isolates in a mouse model by constructing an *ibeB* deletion mutant. To our knowledge, this is the first report of calf meningoencephalitis caused by the rare O161 serotype in the international literature.

## 2. Case Presentation

In winter, an outbreak of fatal neurological disease occurred in neonatal calves on an intensive dairy farm in Shihezi, Xinjiang, China. Calves approximately one week of age successively developed disease, with neurological signs as the predominant presentation. The main clinical signs of the affected calves were elevated body temperature, complete loss of appetite, drooping of the head and ears, an arched back, depression, and emaciation; neurological signs—including head and neck deviation, strabismus, stiff gait, and opisthotonus—were observed, and all affected calves exhibited opisthotonus before death; diarrhea was observed in individual calves. Treatment consisted of empirical antibiotic administration only, without non-steroidal anti-inflammatory drugs, fluid therapy, or electrolytes, and was ineffective. The clinical course lasted 3–7 days, and a total of 13 calves died successively during the outbreak.

**Figure 1:**
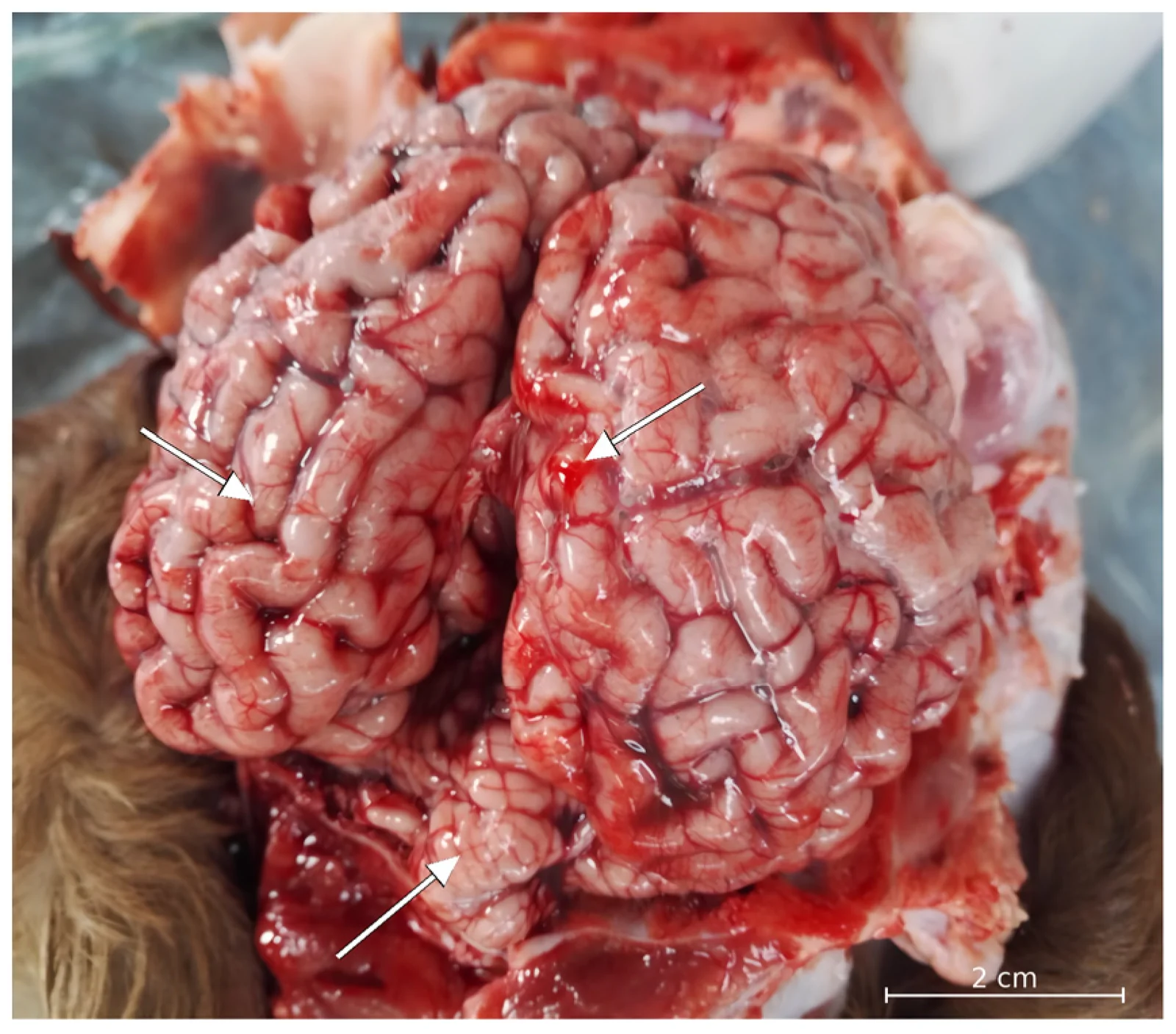
Pathological changes in the brain of a dead calf infected with meningitic *E. coli*: severe meningeal hemorrhage and increased cerebrospinal fluid. Arrows indicate areas of severe meningeal hemorrhage and the accumulation of cerebrospinal fluid. Scale bar = 2 cm.

Necropsy of the dead calves revealed severe hemorrhage of the cerebrum and brainstem and increased cerebrospinal fluid (Figure 1). No obvious gross lesions were observed in other organs, including the heart, lungs, liver, spleen, kidneys, and intestines. Brain and liver tissues from two naturally infected dead calves with neurological signs were aseptically collected and submitted to our laboratory for pathogen identification. Because the outbreak was dominated by neurological disease, no samples were collected for virological examination at the time of the outbreak.

## 3. Laboratory Diagnosis

### 3.1. Bacterial Isolation and Identification

Touch smears of brain and liver tissues stained with Wright’s stain revealed short, straight bacilli under microscopy. Tissues were inoculated into LB medium (tryptone 10 g/L, NaCl 5 g/L, yeast extract 5 g/L) and brain heart infusion (BHI) medium and incubated at 37 °C overnight, followed by streaking onto blood agar, MacConkey agar, and eosin methylene blue (EMB) agar plates at 37 °C for 18–24 h. Five isolates with consistent morphology were obtained: the brain-derived strains CHN-XJ-B1, B2, B3, and B4 and the liver-derived strain CHN-XJ-L; all were Gram-negative short straight bacilli. On LB agar, colonies were round, yellowish-white, smooth, and moist; on MacConkey agar, colonies were round with regular edges; and on EMB agar, colonies were smooth, round, and convex with a black metallic sheen. After incubation at 37 °C for 16–18 h, all isolates exhibited β-hemolysis on blood agar.

Biochemical identification was performed according to *Bergey’s Manual of Systematic Bacteriology* [20]. All five isolates were positive for lactose, glucose, and lysine decarboxylase and negative for oxidase, phenylalanine deaminase, H_2_S, deoxyribonuclease, and citrate (Table 1), consistent with the characteristics of *E. coli*. Two representative strains (CHN-XJ-B1 and CHN-XJ-L) were serotyped by Professor Song Gao (Yangzhou University, Yangzhou, China) and identified as the rare serotype O161. PCR detection showed that all isolates carried the K99 fimbrial gene and the STa enterotoxin gene, whereas F41 was not detected, consistent with the virulence gene profile previously reported for this group of isolates [18].

### 3.2. Antimicrobial Susceptibility Testing

According to the standards of the Clinical and Laboratory Standards Institute (CLSI) [21], the susceptibility of the isolates to 16 antimicrobial agents was determined by the disk diffusion method on BHI agar. All isolates showed identical susceptibility profiles (Table 2): they were resistant to ampicillin, cephalothin, gentamicin, amoxicillin, ciprofloxacin, kanamycin, chloramphenicol, tetracycline, compound sulfamethoxazole, norfloxacin, enrofloxacin, and streptomycin; susceptible to amikacin, cefazolin, and neomycin; and intermediate to polymyxin B. The isolates were thus multidrug-resistant, which is consistent with the failure of empirical antibiotic treatment on the farm.

## 4. Pathogenicity Assessment

### 4.1. Mouse Virulence Test

The median lethal dose (LD_50_) of the two representative strains was determined in a mouse model, with the highly virulent avian pathogenic *E. coli* (APEC) reference strain APEC-XM (kindly provided by Professor Guoqiang Zhu, Yangzhou University, Yangzhou, China) as a control; the mouse model has been widely used for virulence evaluation of ExPEC and APEC [22,23,24]. SPF-grade male Kunming mice (18–22 g) were obtained from Xinjiang Medical University (Urumqi, China) and used at eight weeks of age. Mice were housed under SPF conditions with a 12 h light/dark cycle and free access to food and water, and were randomly allocated to the experimental groups. Animals were monitored at least once daily for clinical signs, including depression, sluggish movement, and dyspnea. Humane endpoints were defined as prostration, severe dyspnea, inability to access food or water, or a body weight loss of more than 20%; mice meeting any of these criteria were euthanized and recorded as deaths, and all surviving mice were euthanized at the end of the observation period. Mice were intraperitoneally injected with 0.2 mL of bacterial suspension containing 5 × 10^4^–5 × 10^8^ CFU (bacterial suspensions were washed and prepared in PBS (NaCl 8 g/L, KCl 0.2 g/L, Na_2_HPO_4_ 1.42 g/L, KH_2_PO_4_ 0.27 g/L), and challenge doses were confirmed by plate counting on LB agar), with 10 mice per dose; negative controls received PBS. Deaths were recorded over 7 days, and the LD_50_ was calculated by the Reed–Muench method [25]. The LD_50_ of CHN-XJ-B1 was independently verified by Kärber’s method in Section 6, with consistent results (see below). The LD_50_ of CHN-XJ-B1 was 9.8 × 10^5^ CFU and that of CHN-XJ-L was 2.3 × 10^6^ CFU, comparable to that of APEC-XM (1.6 × 10^6^ CFU) (Table 3), indicating that the brain-derived isolate was highly virulent. Infected mice showed depression, sluggish movement, and dyspnea; necropsy revealed cerebral hemorrhage and hepatosplenomegaly, and bacteria could be recovered from the brain, liver, and spleen 24 h after infection.

### 4.2. Histopathological Examination

**Figure 2:**
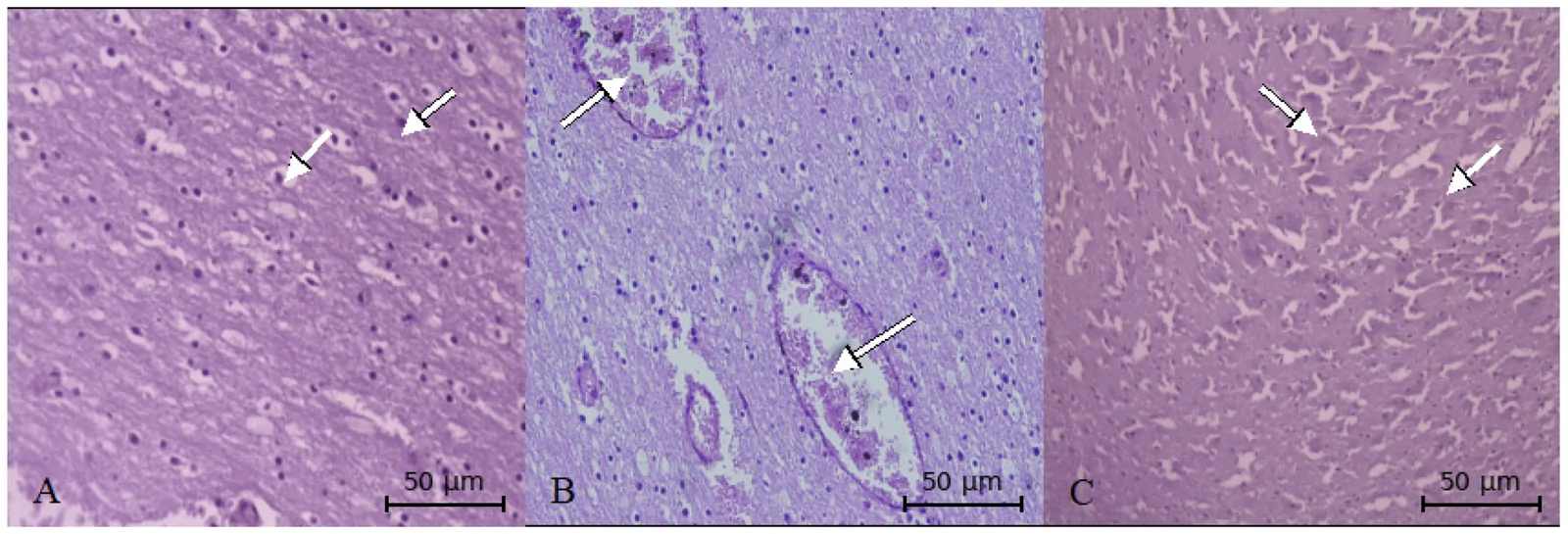
Histopathological changes in brain tissue (HE staining, ×400): (**A**) neuronal degeneration and necrosis; (**B**) cerebral vascular dilation and congestion; (**C**) neutrophilic infiltration of the meninges with degeneration and necrosis, presenting as suppurative encephalitis. Arrowheads indicate the characteristic histological features in each panel. Scale bars = 50 μm.

Brain tissues from naturally infected dead calves were fixed, embedded in paraffin, sectioned, stained with hematoxylin–eosin (HE), and examined microscopically. Severe hemorrhage of the cerebrum and brainstem, increased cerebrospinal fluid, cerebral vascular dilation and congestion, neuronal degeneration and necrosis, and suppurative encephalitis characterized by neutrophilic infiltration of the meninges were observed (Figure 2).

## 5. Detection of the *ibeB* Invasion Gene

**Figure 3:**
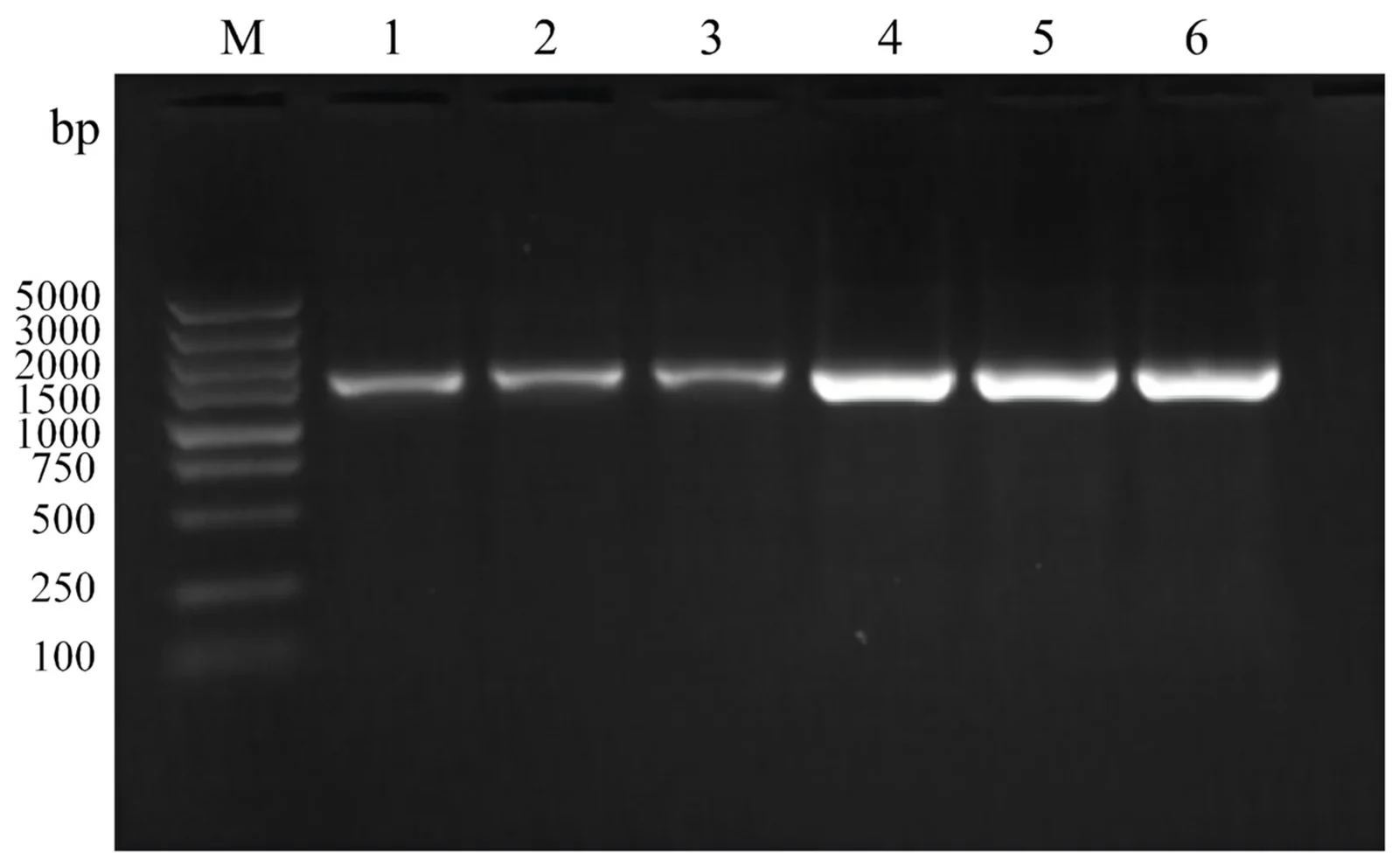
PCR detection of the *ibeB* gene in the isolates. M: DL-5000 DNA Marker; lanes 1–5: CHN-XJ-B1–B4 and CHN-XJ-L; lane 6: APEC-XM.

**Figure 4:**
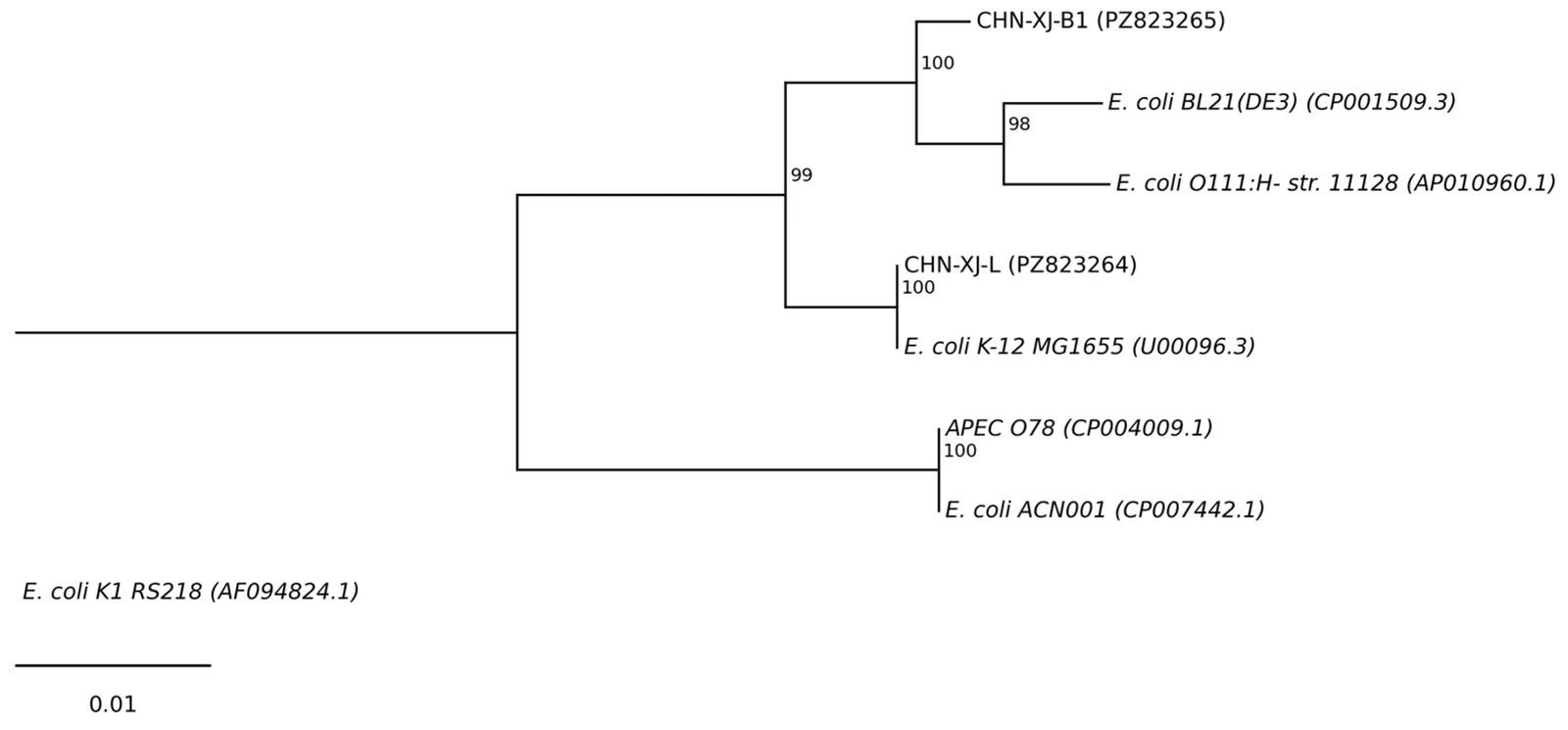
Neighbor-joining phylogenetic tree based on *ibeB* gene nucleotide sequences.

To explore the molecular basis of neuroinvasion by the isolates, *ibeB* gene-specific primers (*ibeB*-F: 5′-ATTTTGTCAATTTTCCTGTCACC-3′; *ibeB*-R: 5′-GCTTTTTTCATTTATTCTCTCCT-3′) were designed with reference to the sequence of the human meningitis *E. coli* K1 strain RS218 (GenBank: AF094824.1) and used for PCR detection. All five isolates yielded target bands of the expected size (~1500 bp) (Figure 3). The amplification products from CHN-XJ-B1 and CHN-XJ-L were cloned (pMD19-T vector) and sequenced: the full-length fragment was 1515 bp and contained a single 1371-bp open reading frame encoding a protein of 457 amino acids (~50 kDa), with a signal peptide predicted at N-terminal residues 1–21; the sequences were deposited in the GenBank database under accession numbers PZ823265 (CHN-XJ-B1) and PZ823264 (CHN-XJ-L), and their sequence features were described in our previous report [17]. Homology analysis of the deposited sequences showed that the ibeB genes of the two isolates shared 98.5% nucleotide identity with each other; they were nearly identical to that of the non-pathogenic *E. coli* K-12 strain MG1655 (CHN-XJ-L: 100% nucleotide and 100% amino acid identity; CHN-XJ-B1: 98.5% and 99.8%, respectively) and shared 96.0–96.4% nucleotide and 95.9–96.1% amino acid identity with RS218. Neighbor-joining (NJ) phylogenetic analysis placed both isolates within the K-12-like clade, together with the avian pathogenic APEC O78 strain and the non-pathogenic reference strains, whereas the human meningitis K1 strain RS218 was positioned on a more distant branch (Figure 4). Bioinformatic analysis predicted IbeB to be a hydrophilic secreted protein without transmembrane helices, with a secondary structure dominated by α-helices and random coils (Figure S2). The complete cloning and bioinformatic analysis of this gene have been reported in our previous study [17] and are briefly summarized here as part of the etiological characterization of this case.

## 6. Construction and Virulence Evaluation of the *ibeB* Deletion Mutant

**Figure 5:**
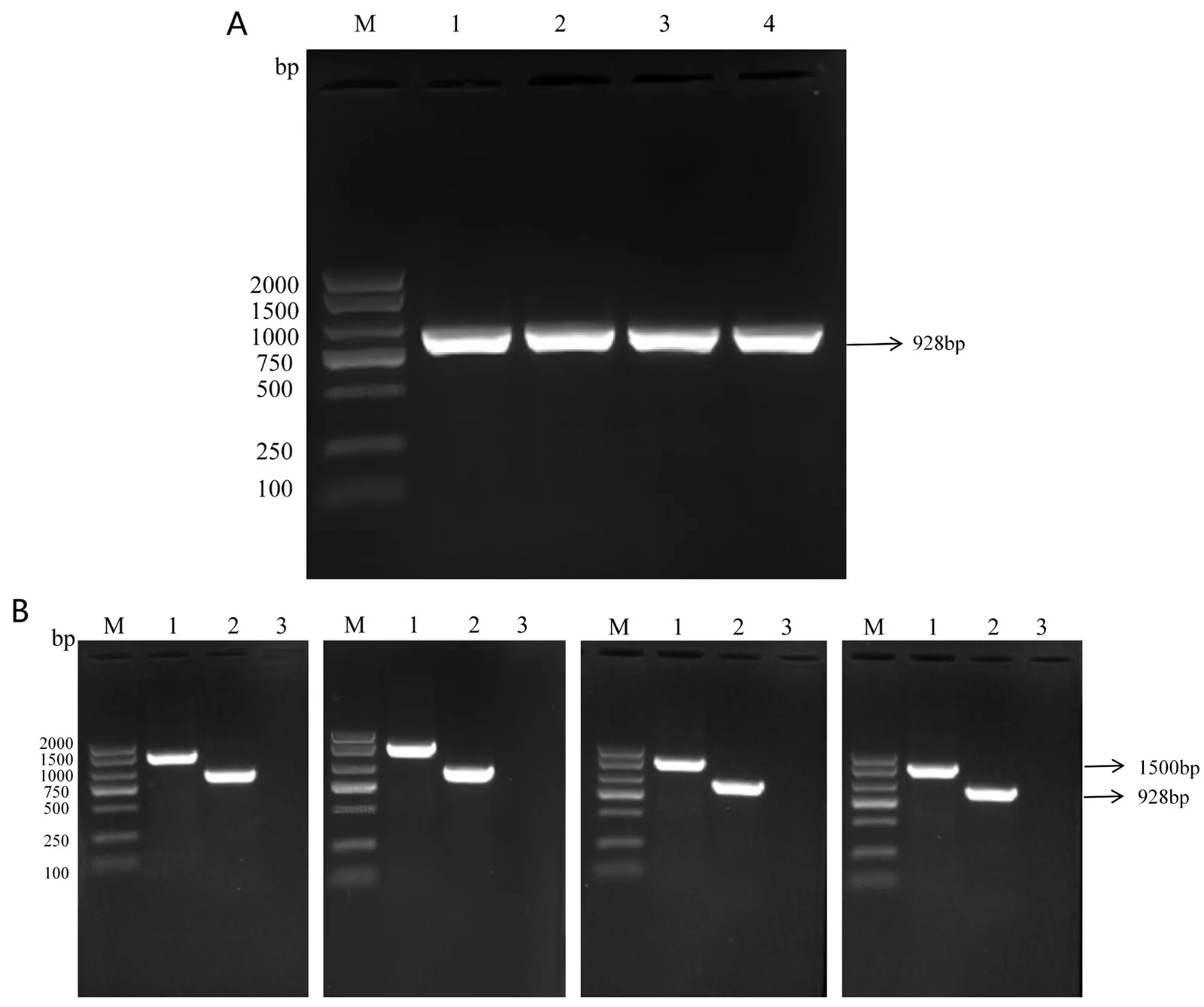
PCR identification of the *ibeB* deletion mutant. (**A**) PCR products of the detection primers (3F/4R) for the mutant CHN-XJ-B1-Δ*ibeB*, 928 bp (M: DL-2000 DNA Marker; lanes 1–4: the mutant); (**B**) genetic stability of the mutant during 30 consecutive passages (checked by PCR every 10 passages; the four panels correspond to passages 10, 20, and 30 of the mutant and the parental strain control, respectively, from left to right) (M: DL-2000 DNA Marker; lane 1: parental strain, ~1500 bp; lane 2: the mutant, 928 bp; lane 3: blank control).

To preliminarily evaluate the role of *ibeB* in the pathogenicity of the isolates, the brain-derived representative strain CHN-XJ-B1 was used as the parental strain, and an *ibeB* deletion mutant, CHN-XJ-B1-Δ*ibeB*, was constructed using the Red/ET homologous recombination system (the detailed construction and identification procedures are described in [19]). After kanamycin selection and excision of the resistance marker with the FLPe plasmid, the mutant yielded only a 928-bp specific fragment by PCR with the detection primers (1500 bp for the parental strain) and failed to grow on tetracycline-containing medium, confirming the complete deletion of *ibeB* (Figure 5A). After 30 consecutive passages without antibiotic pressure (tested every 10 passages), no restoration of the *ibeB* gene was detected, indicating good genetic stability (Figure 5B). The hemolytic properties and in vitro growth ability of the mutant under different pH conditions were not significantly different from those of the parental strain (Figure S1), indicating that *ibeB* deletion does not affect the basic biological characteristics of the strain.

Ten-fold serial dilutions of bacterial suspensions were intraperitoneally injected into 8-week-old mice (0.2 mL per mouse, 10 mice per dose), and deaths were recorded for 10 consecutive days after challenge; the LD_50_ was calculated by Kärber’s method. The LD_50_ of the parental strain CHN-XJ-B1 was 10^6.06^ CFU, essentially consistent with the result determined by the Reed–Muench method in Section 4.1 (9.8 × 10^5^ CFU); the LD_50_ of the mutant CHN-XJ-B1-Δ*ibeB* was 10^7.22^ CFU, approximately one order of magnitude higher than that of the parental strain (Table 4), suggesting that deletion of *ibeB* attenuated virulence.

**Figure 6:**
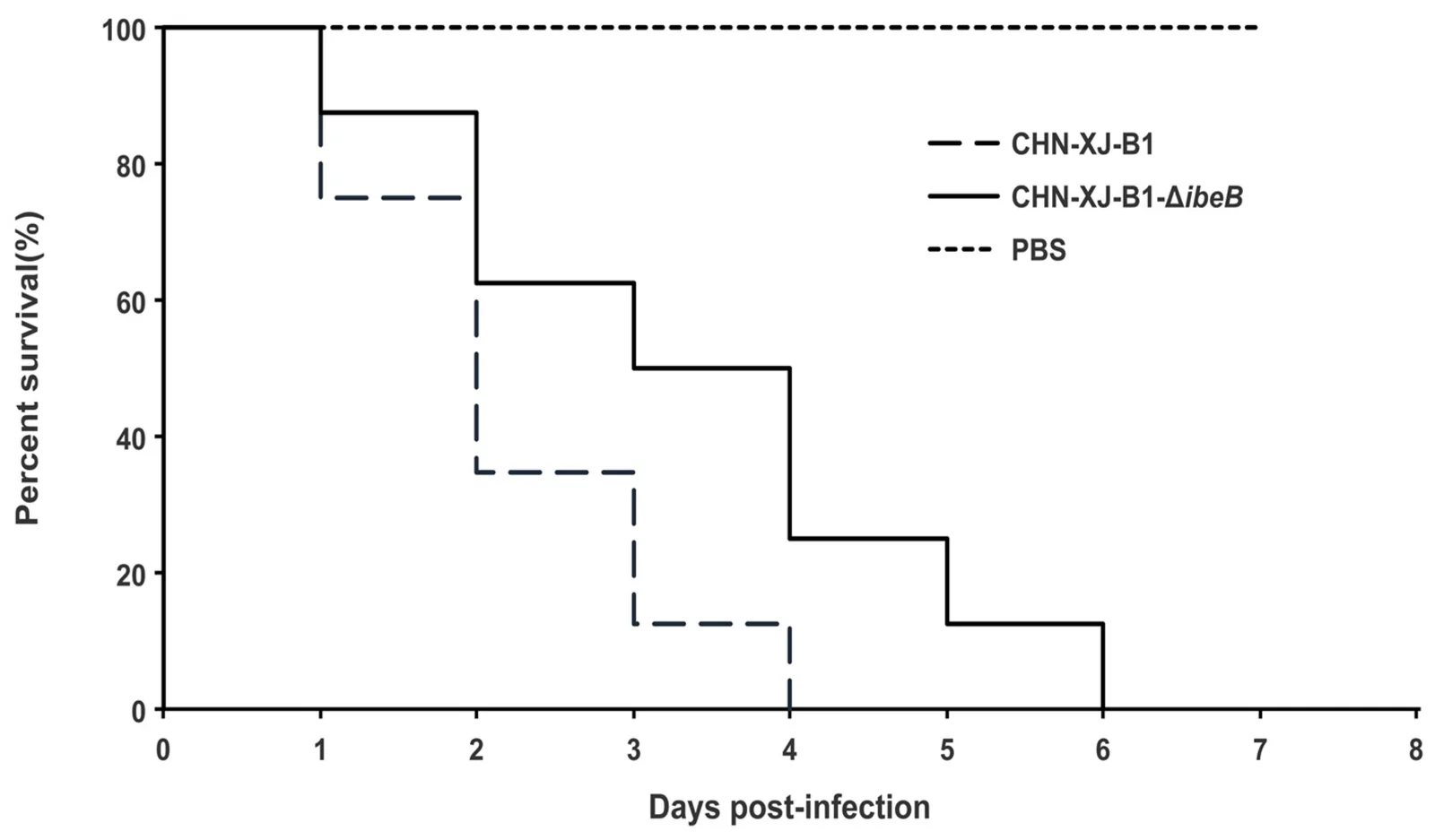
Kaplan–Meier survival curves of mice infected with the parental strain, the *ibeB* deletion mutant (10^7^ CFU per mouse, intraperitoneal injection), or PBS (negative control).

**Figure 7:**
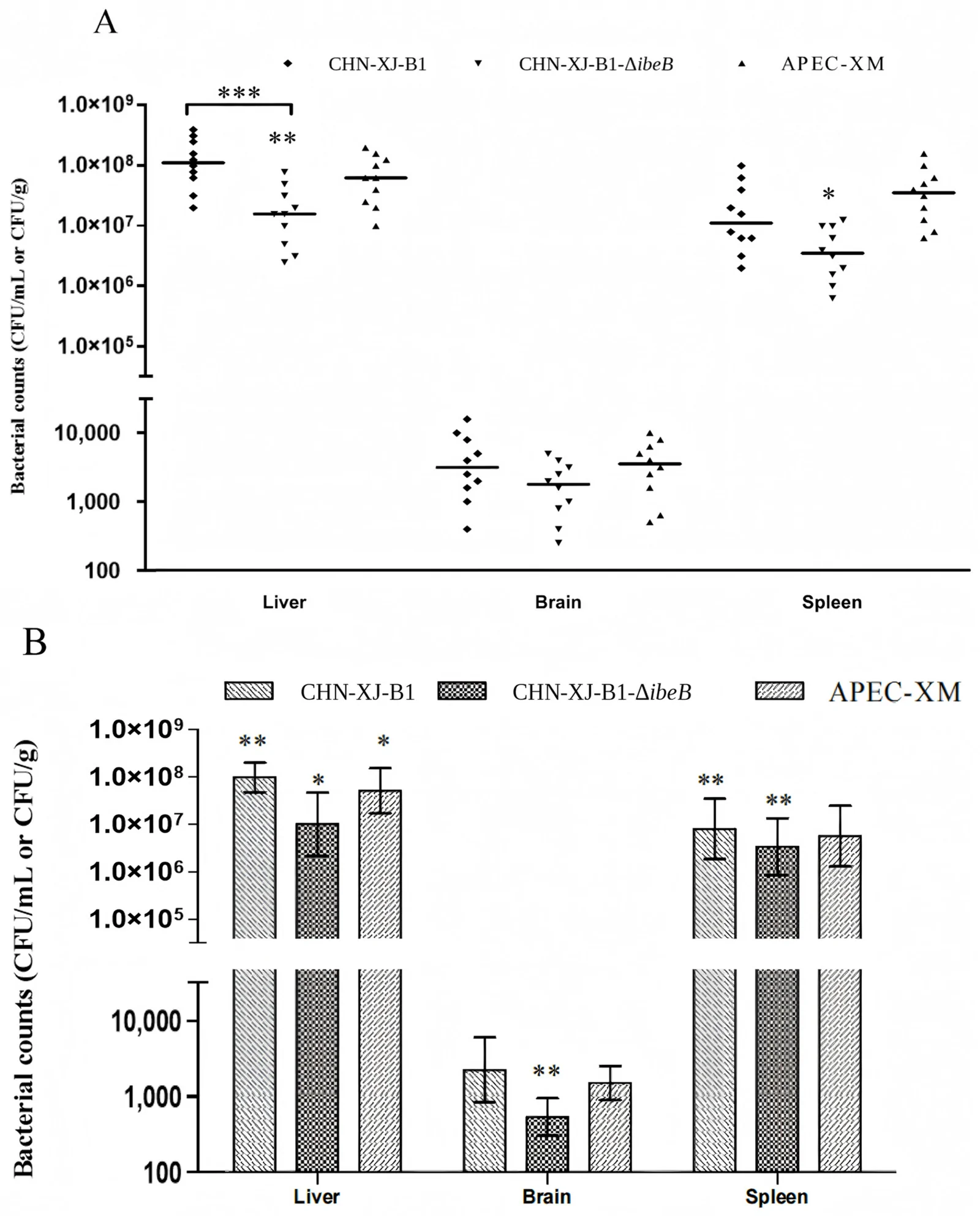
Bacterial loads in the liver, spleen, and brain of mice infected with the parental strain CHN-XJ-B1, the mutant CHN-XJ-B1-Δ*ibeB*, and APEC-XM at 24 h (**A**) and 48 h (**B**) post-infection (intraperitoneal injection of approximately 3.4 × 10^6^ CFU per mouse; * *p* < 0.05, ** *p* < 0.01, *** *p* < 0.001). In panel (**A**), each symbol represents an individual mouse and horizontal lines indicate the median; in panel (**B**), bars represent the mean ± SD. In panel (**B**), asterisks above the bars indicate significant differences compared with the parental strain; in particular, the double asterisk above the brain bar indicates a significant difference between the mutant and the parental strain at 48 h post-infection, whereas the difference in brain bacterial loads at 24 h (panel (**A**)) was not significant.

Mice were intraperitoneally injected with 10^7^ CFU, and deaths were recorded daily to plot Kaplan–Meier survival curves: the mean survival time of mice in the mutant group (3.38 days) was significantly longer than that in the parental strain group (2.25 days) (*p* < 0.05), and no deaths occurred in the PBS control group (Figure 6). Additional mice were randomly divided into three groups (parental strain CHN-XJ-B1, mutant CHN-XJ-B1-Δ*ibeB*, and APEC-XM control; 30 mice per group) and intraperitoneally injected with bacterial suspensions cultured to OD_600_ ≈ 0.30 (0.2 mL per mouse, approximately 3.4 × 10^6^ CFU). At 24, 48, and 72 h post-infection, 10 mice per group were euthanized by cervical dislocation; liver, spleen, and brain tissues were aseptically collected, homogenized, serially diluted 10-fold, and plated on MacConkey agar for counting. The results showed that the bacterial loads in the liver of mice in the mutant group were significantly lower than those in the parental strain group at 24 h (*p* < 0.01) and 48 h (*p* < 0.05), and the bacterial loads in the spleen were also significantly reduced at 24 h (*p* < 0.05) and 48 h (*p* < 0.01); the brain bacterial loads of all groups were around 10^3^ CFU/mL; the brain loads did not differ significantly between the mutant and parental strain groups at 24 h, but were significantly lower in the mutant group at 48 h (*p* < 0.01) (Figure 7). Because most mice in the parental strain and APEC-XM groups developed severe clinical signs and died after 48 h, the 72-h data were not included in the analysis. Mice inoculated with the parental strain and APEC-XM showed obvious hepatosplenomegaly, whereas those inoculated with the mutant showed milder enlargement of the liver and spleen. Bacteria could be recovered from the liver, spleen, and brain tissues of infected mice; PCR detection showed that the bacteria recovered from the parental strain group yielded a 1500-bp fragment and those from the mutant group yielded a 928-bp fragment, consistent with the inoculated strains. Survival curves were compared using the log-rank (Mantel–Cox) test, and bacterial loads were compared by one-way ANOVA followed by Tukey’s multiple comparisons test; all statistical analyses were performed with GraphPad Prism 9 (GraphPad Software, San Diego, CA, USA), and *p* < 0.05 was considered statistically significant.

## 7. Discussion

Colibacillosis in neonatal calves mostly presents as the enteric or septicemic form, and the meningitic form is rarely reported. This outbreak was caused by *E. coli* of the rare O161 serotype: the common serotypes of pathogenic *E. coli* from calves in northern Xinjiang are O78, O50, and O114 [26], whereas O161 had never been associated with calf meningoencephalitis before. Following this outbreak, a dairy farm in Argentina reported cases of meningitis and septicemia in calves, from which multidrug-resistant *E. coli* of phylogroups A and C was isolated [15]; more recently, a whole-genome analysis of a capsule-deficient NMEC strain (O18ab:H14 serotype, ST1434 sequence type) causing acute meningitis in calves was reported in China [16]. In China, encephalitis-associated *E. coli* has also been repeatedly reported in the Shihezi region, including the isolation and identification, antimicrobial resistance, and virulence gene detection of calf encephalitis *E. coli* [27], pathogenicity studies in cell and mouse infection models [28], and our laboratory’s earlier isolation, identification, and histopathological observation of calf encephalitis *E. coli* [18]. The serotype in the present case (O161) differs from those in the above reports, further expanding the known serotype spectrum of bovine meningitis-causing *E. coli*. Notably, no gross lesions were observed in organs other than the brain, yet *E. coli* was isolated from the liver (strain CHN-XJ-L); this is consistent with the pathogenesis of the meningitic form of colibacillosis, in which bacteria disseminate hematogenously and the liver, as a major blood-filtering organ, commonly harbours bacteria in bacteraemic animals even in the absence of gross pathological changes.

The resistance profile of the isolates provides a reasonable explanation for the treatment failure on the farm. The isolates were resistant to 12 of the 16 tested drugs, covering the classes most commonly used on dairy farms (penicillins, aminoglycosides, quinolones, tetracyclines, and sulfonamides). Over the past decade, the prevalence of multidrug resistance among bovine *E. coli* has increased significantly worldwide: the latest surveillance in Italy showed that the proportion of multidrug-resistant strains among intestinal *E. coli* of dairy and veal calves exceeded 75% [29]; New Zealand reported high detection rates of tetracycline- and streptomycin-resistant and multidrug-resistant *E. coli* in dairy calves [30]; and the United States found extensive co-existence of resistance genes and virulence genes in highly resistant isolates from beef calves [31]. The multidrug resistance profile of our isolates is consistent with these global trends. For veterinary clinical practice, this case suggests that empirical medication is unreliable for calves with suspected bacterial meningitis and that drugs should be selected on the basis of susceptibility testing; according to our results, amikacin, cefazolin, or neomycin would be reasonable candidate drugs. Moreover, because most antimicrobial agents have difficulty crossing the blood–brain barrier, the treatment of bacterial meningitis is inherently more difficult than that of other infections, making early and accurate diagnosis crucial. In addition, the treatment on the farm consisted of antibiotics alone, without supportive therapy such as non-steroidal anti-inflammatory drugs or fluid and electrolyte supplementation, which may also have contributed to the high case-fatality rate.

The isolates possess both enteric pathogenic weaponry (K99 fimbriae and STa enterotoxin, with F41 absent—a typical configuration of enterotoxigenic *E. coli* causing diarrhea in calves [32]) and the neuroinvasion determinant *ibeB*, suggesting that they belong to an ExPEC phenotype with both intestinal and extraintestinal pathogenic potential. *ibeB* is associated with *E. coli* isolated from the cerebrospinal fluid of meningitis patients but is absent from commensal strains [33]; IbeB participates in the invasion and pathogenicity of avian pathogenic *E. coli* [22], and avian strains themselves can cause meningitis in mammalian models [34]. In this study, we functionally verified this association by constructing an *ibeB* deletion mutant via Red/ET homologous recombination (Section 6) [19]: the LD_50_ of the mutant in mice was approximately one order of magnitude higher than that of the parental strain, the survival time of infected mice was significantly prolonged, and the bacterial loads in the liver and spleen were significantly reduced, directly supporting a role for *ibeB* in the pathogenicity of this strain. Notably, the brain bacterial load of the mutant did not decrease significantly at 24 h post-infection but was significantly reduced at 48 h, suggesting that *ibeB* acts mainly during hematogenous dissemination and subsequent persistence in the brain rather than in the initial crossing of the blood–brain barrier itself, a hypothesis that awaits further verification in cell invasion models. The *ibeB* of our isolates shared 98.5–100% nucleotide identity with that of the non-pathogenic *E. coli* K-12 strain and approximately 96% with the human meningitis K1 strain RS218, indicating that ibeB is more widely distributed among *E. coli* lineages than previously recognized. This near-identity to a non-pathogenic K-12 ibeB variant, together with its divergence from that of the human meningitis strain RS218, may limit its invasion-promoting activity at the blood–brain barrier, which is consistent with the delayed reduction in brain bacterial loads observed only at 48 h post-infection; nevertheless, its deletion increased the LD_50_ of our isolate by approximately one order of magnitude, supporting a functional role of this K-12-like ibeB variant in systemic pathogenicity. These findings suggest that bovine and human meningitis-causing *E. coli* may share similar invasion mechanisms.

Limitations of this report should be noted: First, the investigation involved only a single farm, and because the outbreak was dominated by neurological disease, no samples were collected for virological examination; thus, the involvement of viral pathogens could not be excluded, although the bacterial etiology is strongly supported by the consistent isolation of *E. coli* from the brain and liver of both necropsied calves, the characteristic suppurative (neutrophilic) meningoencephalitis, and the reproduction of lethal disease in experimentally infected mice. Second, only O-antigen serotyping was performed, and the H-antigen and capsular (K) antigens of the isolates were not characterized; since the K1 capsule is a hallmark of classical NMEC strains, the capsular type of these O161 isolates remains to be determined, and whole-genome sequencing, MLST, and PCR screening for capsule-encoding loci (e.g., *neuC* and *kpsMT*) are planned as follow-up work. Third, a complemented strain (C-Δ*ibeB*) was not constructed; however, the Δ*ibeB* mutant showed no significant in vitro growth defect compared with the parental strain (Figure S1); the Red/ET homologous recombination used here generates a scarless, in-frame deletion, which minimizes the risk of polar effects on downstream genes, and the attenuated phenotype of Δ*ibeB* was consistently observed across independent experiments, including the LD_50_ determination, the survival analysis, and the bacterial-load quantification; nevertheless, polar effects or unintended secondary mutations cannot be formally excluded, and complementation analysis will be performed in future work. Fourth, the role of *ibeB* in crossing the blood–brain barrier was not verified in a cell invasion model. Apart from this case, the O161 serotype has not been reported in association with calf meningoencephalitis, and the pathogenicity data presented here remain valuable for current surveillance and control. Field evidence indicates that vaccinating cows with an enterotoxigenic *E. coli* vaccine improves calf survival [35], and evaluating IbeB as a vaccine target against meningitis-causing *E. coli* is likewise worth exploring.

## 8. Conclusions

This study reports an outbreak of fatal meningoencephalitis in neonatal calves caused by *E. coli* of the rare O161 serotype (carrying the K99 and STa virulence genes and the *ibeB* invasion gene). The isolates were highly virulent in mice and multidrug-resistant, which explains the failure of empirical antibiotic treatment on the farm; virulence evaluation of the *ibeB* deletion mutant indicated that *ibeB* contributes to the systemic dissemination and tissue colonization of this strain. To our knowledge, this is the first report of calf meningoencephalitis caused by O161 *E. coli* in the international literature. Clinicians should be alert to the meningitic form of colibacillosis in neonatal calves and select antimicrobial agents on the basis of susceptibility testing results.

## Figures and Tables

**Table 1:** Biochemical reactions of the isolated strains.

Biochemical Reaction	CHN-XJ-B1	CHN-XJ-B2	CHN-XJ-B3	CHN-XJ-B4	CHN-XJ-L
Oxidase	−	−	−	−	−
Phenylalanine deaminase	−	−	−	−	−
H_2_S	−	−	−	−	−
Deoxyribonuclease	−	−	−	−	−
Citrate (Simmons)	−	−	−	−	−
D-Adonitol	−	−	+	−	+
Lysine decarboxylase	+	+	+	+	+
Lactose	+	+	+	+	+
Glucose	+	+	+	+	+
Cellobiose	−	−	+	−	+

Note: “+”, positive; “−”, negative.

**Table 2:** Results of the antimicrobial susceptibility tests.

Antibiotic	Content (μg/Disk)	Inhibition Zone Diameter (mm)	Result
Ampicillin	10	0 mm	R
Cephalothin	5	0 mm	R
Gentamicin	10	0 mm	R
Amikacin	30	18	S
Amoxicillin	50	0 mm	R
Ciprofloxacin	5	0 mm	R
Kanamycin	30	12	R
Chloramphenicol	30	0 mm	R
Polymyxin B	300	16	I
Tetracycline	30	0 mm	R
Compound sulfamethoxazole	1.25/23.75	0 mm	R
Norfloxacin	10	0 mm	R
Enrofloxacin	5	0 mm	R
Streptomycin	10	0 mm	R
Cefazolin	30	18	S
Neomycin	30	20	S

Note: “0 mm” indicates that no inhibition zone was observed; S, susceptible; I, intermediate; R, resistant.

**Table 3:** Calculation of the LD_50_ of different bacterial isolates.

Challenge Dose (CFU)	CHN-XJ-B1	CHN-XJ-L	APEC-XM
5 × 10^8^	10/10	10/10	10/10
5 × 10^7^	10/10	10/10	10/10
5 × 10^6^	10/10	7/10	8/10
5 × 10^5^	3/10	1/10	2/10
5 × 10^4^	0/10	0/10	0/10
LD_50_ value (CFU)	9.8 × 10^5^	2.3 × 10^6^	1.6 × 10^6^

Note: values are the number of dead mice/total number of mice.

**Table 4:** Comparison of the LD_50_ of the parental strain CHN-XJ-B1 and the deletion mutant CHN-XJ-B1-Δ*ibeB* in mice (Kärber’s method).

Challenge Dose (CFU)	CHN-XJ-B1 (Parental Strain)	Challenge Dose (CFU)	CHN-XJ-B1-ΔibeB (Mutant)
2.3 × 10^9^	10/10	5.2 × 10^9^	10/10
2.3 × 10^8^	10/10	5.2 × 10^8^	9/10
2.3 × 10^7^	10/10	5.2 × 10^7^	7/10
2.3 × 10^6^	7/10	5.2 × 10^6^	4/10
2.3 × 10^5^	1/10	5.2 × 10^5^	0/10
LD_50_ value (CFU)	10^6.06^	LD_50_ value (CFU)	10^7.22^

Note: values are the number of dead mice/total number of mice.

## Data Availability

The data presented in this study are openly available in GenBank at https://www.ncbi.nlm.nih.gov/genbank/ (accessed on 29 August 2026), accession numbers PZ823265 and PZ823264.

## Supplementary Materials

The following supporting information can be downloaded at: https://www.mdpi.com/article/10.3390/vetsci13101037/s1, Figure S1: In vitro growth curves of the parental strain CHN-XJ-B1 and the deletion mutant CHN-XJ-B1-Δ*ibeB* under different pH conditions ((A) pH 3.0; (B) pH 5.0; (C) pH 7.0; (D) pH 9.0); Figure S2: Bioinformatic analysis of the IbeB protein ((A) transmembrane region prediction; (B) hydrophobicity analysis; (C) and (D) secondary structure predictions for CHN-XJ-B1 and CHN-XJ-L, respectively; (E) comprehensive protein sequence analysis; (F) three-dimensional structure prediction).

## Abbreviations

The following abbreviations are used in this manuscript:

NMEC	Neonatal meningitis-causing *Escherichia coli*
ExPEC	Extraintestinal pathogenic *Escherichia coli*
APEC	Avian pathogenic *Escherichia coli*
BBB	Blood–brain barrier
BMEC	Brain microvascular endothelial cell
BHI	Brain heart infusion
EMB	Eosin methylene blue
LB	Luria–Bertani
PBS	Phosphate-buffered saline
LD_50_	Median lethal dose
CFU	Colony-forming unit
CLSI	Clinical and Laboratory Standards Institute
HE	Hematoxylin–eosin
NJ	Neighbor-joining
